# Prevalence of paratuberculosis in organized and unorganized dairy cattle herds in West Bengal, India

**DOI:** 10.14202/vetworld.2017.574-579

**Published:** 2017-06-02

**Authors:** Jitendrakumar M. Bhutediya, Premanshu Dandapat, Arijit Chakrabarty, Ratan Das, Pramod Kumar Nanda, Samiran Bandyopadhyay, Tapas Kumar Biswas

**Affiliations:** Eastern Regional Station, ICAR-Indian Veterinary Research Institute, Kolkata - 700 037, West Bengal, India

**Keywords:** cattle, India, Johne’s disease, paratuberculosis, prevalence, West Bengal

## Abstract

**Aim::**

The aim of this study was to determine the prevalence pattern of *Mycobacterium avium* subsp. *paratuberculosis*, the causative agent of paratuberculosis or Johne’s disease, in unorganized as well as organized cattle herds in West Bengal.

**Materials and Methods::**

Four organized cattle farms with identical management practice in Nadia (n=3) and South 24 Parganas (n=1) districts and three unorganized cattle herds, one each from three districts, namely, Burdwan, North 24 Parganas, and Purba Midnapur, were selected randomly and screened for paratuberculosis by delayed-type hypersensitivity (DTH) and enzyme-linked immunosorbent assay (ELISA).

**Results::**

Of 191 animals tested by DTH, 57 (29.8%) were found to be positive in comparison to 72 (37.7%) by ELISA. In organized farms, seropositivity varied from 13.3% to 53.1%, whereas in unorganized sector, it ranged from 5% to 6.7% with one area having exceptionally high prevalence, i.e. 53.3%. The range of positivity detected by DTH both in organized farms and backyard sectors varied from 0% to 46.7%. By employing both DTH and ELISA together, the positivity of animals in organized and unorganized herds was 19.9% and 8%, respectively.

**Conclusion::**

The results indicate that animals in organized farms are much more prone to paratuberculosis than others. For screening the herd, both DTH and ELISA should be used simultaneously to increase the test sensitivity in order to minimize its further spread adopting control programs.

## Introduction

Paratuberculosis, caused by *Mycobacterium avium* subsp. *paratuberculosis* (MAP), is one of the most widespread bacterial diseases capable of infecting a wide array of animal species including ruminants in agriculturally developed countries [1,2]. The Office International des Epizooties (OIE) considered paratuberculosis as a disease of major global importance and categorized it as a List B transmissible disease [3]. Under natural conditions, the disease is spread by ingestion of MAP from the contaminated environment [4], practice of feeding pooled colostrum containing viable bacteria to calves [5] or contamination of the pasture with infected animal feces. MAP has the potential to cause public health implications also, as cells of these organisms have the ability to survive pasteurization and transmission to human can occur through raw milk, meat, and even contact with animal [6-8]. Due to these, research to study the prevalence pattern of MAP in different countries has gained momentum over the past few years.

Economic losses to the dairy cattle industry due to the paratuberculosis are mainly associated with decreased milk production, weaning weight loss in young calves, reduced slaughter value thereby prompting to take action for early culling and trade restriction [9-11]. On an average, the economic loss caused by bovine paratuberculosis has been estimated to be higher than those for other bovine diseases such as bovine viral diarrhea, enzootic bovine leukosis, and neosporosis [12].

As far as India is concerned, paratuberculosis, otherwise known as Johne’s disease (JD), is endemic in domestic livestock. Although this was first reported at Hisar, India way back in 1913, national estimates on the prevalence of MAP are still not available [13-15]. There are a few reports on the prevalence of JD in cattle in western, northern, and southern states of India such as Gujarat [16], Punjab [17], and Tamil Nadu [18]. Its prevalence in small ruminants has also been studied in states such as Madhya Pradesh [19], Maharashtra [20], Uttar Pradesh [21], and Rajasthan [22]. Way back in the 1960s, 1% prevalence of JD was reported in West Bengal on the basis of tissue smear examination of slaughterhouse specimens [23]. Except this, hardly any data is available on the prevalence of paratuberculosis in cattle population in eastern India, including West Bengal.

Although various techniques are employed for the diagnosis of JD [24], herd screening test is usually conducted to identify the animals as actually infected or uninfected. However, none of the test methods provide accurate results due to various limitations. Given the above, the present study was undertaken to employ two primary screening tests, namely, delayed-type hypersensitivity (DTH) and enzyme-linked immunosorbent assay (ELISA) to investigate the prevalence pattern of MAP within dairy herds, both in organized farms and unorganized sectors in southern Gangetic delta of West Bengal.

## Materials and Methods

### Ethical approval

During this study, all animal handling procedures were performed after obtaining prior approval from the Institute Animal Ethics Committee. All applicable guidelines for the care and use of animals were also followed.

### Selection of area and farms/herds

The study was carried out to find the prevalence of MAP infection in cattle (n=191) reared in both organized (4) and unorganized dairy herds (3) covering five districts, namely, Burdwan, Purba Midnapur, Nadia, North 24 Parganas, and South 24 Parganas of West Bengal (Table-1). Of a total 16 organized farms in these districts of West Bengal, 4 farms were selected for the study through simple random sampling. In each organized farm, about 10% of the total animals were selected through systematic random sampling method. The animals from surrounding areas were selected following random sampling procedure to get first-hand information on percent positivity (PP) of paratuberculosis in the backyard sector. The representative animals, selected randomly from each of these herds, were screened by DTH reaction as well as ELISA for paratuberculosis.

**Table-1:** Details of cattle selected for screening of paratuberculosis by DTH and ELISA.

Organized/unorganized farm	District in West Bengal	Number of cattle screened by DTH and ELISA
Organized Farm I	Nadia	96
Organized Farm II	South 24 Parganas	15
Organized Farm III	Nadia	15
Organized Farm IV	Nadia	15
Unorganized Farm I	Burdwan	20
Unorganized Farm II	North 24 Parganas	15
Unorganized Farm III	Purba Midnapur	15

DTH=Delayed-type hypersensitivity, ELISA=Enzyme linked immunosorbent assay

### Testing of cattle by DTH reaction (Johnin test)

Testing of cattle by DTH reaction (Johnin test) was carried out following the protocol of OIE [25]. In brief, the skin was shaven on the side of the middle-third of the neck to which 0.1 ml of Johnin-purified protein derivative (PPD) (3000 IU/dose, MAP MTCC 19698, IVRI, Izatnagar) was injected intradermally. A correct injection was confirmed by palpating a small pea-like swelling at each site of injection. The skin thickness was measured using Vernier caliper before and after 72±2 h of inoculation. The test results of the intradermal tuberculin test were interpreted as per the OIE standards [26]. An increase in skin thickness of over 2 mm, 72 h after inoculation, was considered as positive to DTH.

### Screening of serum by ELISA

The blood samples were collected from cattle before skin testing for DTH. A minimum of 5 ml blood sample was taken from the jugular vein of each animal using 18 gauge needles and serum collection tubes (vacutainer, serum REF 367815 Becton Dickinson, New Jersey, USA). Care was taken to put the tubes in a slanting position for 30 min without any disturbances during clotting. Clotted blood samples were kept in the refrigerator at 4°C for 5-6 h. Serum samples were harvested in fresh and sterilized vials and stored at −20°C until further use.

Serum ELISA was carried out by the *Mycobacterium paratuberculosis* test kit for cattle PARACHEK^®^ 2 (Prionics, USA) as per manufacturer’s instruction. The test was considered valid if mean corrected value of positive control (PC) was >0.500 (i.e., OD_PC_ >0.500) and 5 times more than the corrected value of negative control (NC) (OD_PC_/OD_NC_> 5). Based on this, the calculated value of serum samples above or equal to the cut-off of 15 PP were considered positive, whereas cut-off below 15 PP were considered negative. The PP of each sample based on the OD values was calculated by following formula:


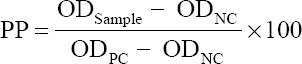


### Statistical analysis

The statistical software (SPSS 20.0 version) was used for analysis of the data, whereas Microsoft Word and Excel were used to generate tables and graphs. A Chi-square test was conducted to compare the association between the prevalence of paratuberculosis based on either single diagnostic test (DTH or ELISA) or combined testing by DTH and ELISA and farming system as well as herds within each farming system.

## Results

### Testing of cattle by DTH and ELISA

**Figure-1:**
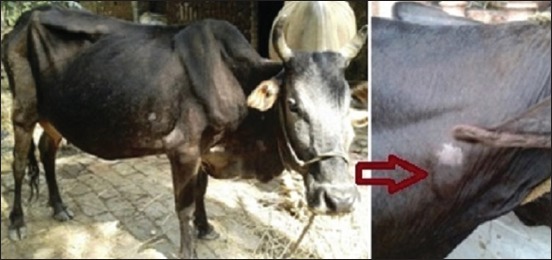
A case of positive Johnin test (delayed-type hypersensitivity) in cattle reared in unorganized farm.

**Figure-2:**
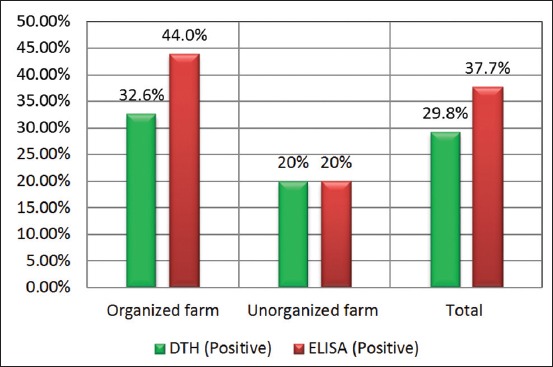
Prevalence of paratuberculosis in organized and unorganized dairy cattle farms in representative districts of West Bengal by delayed-type hypersensitivity and enzyme-linked immunosorbent assay.

The farm-wise PP of paratuberculosis in cattle by DTH and ELISA are depicted in Table-2. Of 191 animals tested by DTH (Figure-1), 57 (29.8%) and 134 (70.2%) were found to be positive and negative, respectively for paratuberculosis, in comparison to 72 (37.7%) positive and 119 (62.3%) negative to ELISA (Table-2 and Figure-2). The seropositivity in organized farms varied from 13.3% to 53.1%, whereas in the unorganized sector, it ranged from 5% to 6.7% with one area having exceptionally high prevalence, i.e. 53.3%. The range of positivity detected by DTH in both organized farms and backyard sectors was found to be similar, that is, from 0% to 46.7% (Table-2). The level of positivity based on corresponding Chi-square value, p-value and likelihood ratio in different herds within organized and unorganized farm using single diagnostic test (DTH or ELISA) differed significantly and is presented in Table-2.

**Table-2:** Screening of herds by DTH and ELISA for prevalence of paratuberculosis in organized and unorganized farms.

Farm	Prevalence of paratuberculosis (based on both DTH and ELISA)										

	DTH					ELISA				
	
	DTH positive (%)	DTH negative (%)	Chi-square value	p	Likelihood ratio	ELISA positive (%)	ELISA negative (%)	Chi-square value	p	Likelihood ratio
Organized farm										
Herd-I	34 (35.4)	62 (64.6)	12.335	0.015	16.44	51 (53.1)	45 (46.9)	23.897	0.001	25.179
Herd-II	0 (0)	15 (100)				2 (13.3)	13 (86.7)			
Herd-III	5 (33.3)	10 (66.7)				3 (20.0)	12 (80.0)			
Herd-IV	7 (46.7)	8 (53.3)				6 (40.0)	9 (60.0)			
Total	46 (32.6)	95 (67.4)				62 (44.0)	79 (56.0)			
Unorganized farm										
Herd-I	0 (0.0)	20 (100)	11.846	0.008	17.286	1 (5.0)	19 (95.0)	18.296	0.001	22.722
Herd-II	3 (20.0)	12 (80.0)			1 (6.7)	14 (93.3)				
Herd-III	7 (46.7)	8 (53.3)			8 (53.3)	7 (46.7)				
Total	10 (20)	40 (80)			10 (20)	40 (80)				
Grand total	57 (29.8)	134 (70.2)	2.839	0.092	2.986	72 (37.7)	119 (62.3)	13.001	0.001	14.234

DTH=Delayed-type hypersensitivity, ELISA=Enzyme linked immunosorbent assay

Likewise, the test results of combined diagnostic test (DTH and ELISA) in four combinations, *viz*., DTH positive and ELISA positive; DTH negative and ELISA negative; DTH positive and ELISA negative, and DTH negative and ELISA positive are given in Table-3. In organized farms, of 141 cattle, 28 (19.9%) were tested positive to both DTH and ELISA while 60 (42.5%) were negative to both the tests. Further, 35 (24.8%) cattle were found to be positive to ELISA and negative to DTH and 18 (12.8%) were tested negative to ELISA and positive to intradermal Johnin test (Table-3). In backyard sector, 4 (8%) of 50 cattle tested positive to both Johnin and ELISA tests and 36 (72%) were found to be negative to both the tests. A total of 5 (10%) cattle were tested positive to ELISA but negative to DTH. A same number of animals (n=5) were also negative to ELISA and positive to Johnin test (Table-3).

**Table-3:** Prevalence of paratuberculosis in organized and unorganized cattle herds employing combined tests (DTH and ELISA).

Farming system	Animals tested				Total

	DTH positive, ELISA positive (%)	DTH negative, ELISA negative (%)	DTH positive, ELISA negative (%)	DTH negative, ELISA positive (%)	
Organized I	24 (25.0)	35 (36.5)	10 (10.4)	27 (28.1)	96
Organized II	0 (0)	12 (80)	0 (0)	3 (20)	15
Organized III	1 (6.7)	8 (53.3)	4 (26.7)	2 (13.3)	15
Organized IV	3 (20)	5 (33.3)	4 (26.7)	3 (20)	15
Total	28 (19.9)	60 (42.5)	18 (12.8)	35 (24.8)	141
Unorganized I	0 (0)	19 (95.0)	0 (0)	1 (5.0)	20
Unorganized II	0 (0)	11 (73.3)	3 (20.0)	1 (6.7)	15
Unorganized III	4 (26.7)	6 (40.0)	2 (13.3)	3 (20.0)	15
Total	4 (8.0)	36 (72)	5 (10.0)	5 (10.0)	50
Grand total	32 (16.8)	96 (50.3)	23 (12.0)	40 (20.9)	191

DTH=Delayed-type hypersensitivity, ELISA=Enzyme linked immunosorbent assay

Employing Chi-square tests for association between farming system or herds and prevalence of paratuberculosis, the combined result (DTH and ELISA) revealed a significant (p≤0.003) difference in the prevalence of paratuberculosis among animals in organized farms compared to unorganized farms. Further, the likelihood ratio of occurrence of paratuberculosis in organized and unorganized farms by DTH was lower (16.44 vs. 17.286) than ELISA (25.179 vs. 22.722), respectively.

## Discussion

Paratuberculosis or JD, an intestinal granulomatous infection caused by MAP, is mostly found among domestic ruminants including cattle, buffalo, sheep, goat, and camelids as well as wild ruminants (cervids) [26]. The disease causes huge economic loss to the dairy cattle industry which is estimated to be about $200 per infected cow per year in herds with at least 10% prevalence [10,27,28]. MAP is also important due to its zoonotic potential and is believed to be a pathogenic agent for the granulomatous ulceroconstrictive disease of the ileocecal region, particularly Crohn’s disease in human [1,27,29,30]. Screening of herd to know the prevalence pattern of MAP affecting different ruminant species is, therefore, an essential requirement to develop diagnostic methods and immunoprophylaxis for control and prevention of JD. Although JD is reported to be highly endemic in India [15,31], no study has yet been conducted in eastern regions to find its status in cattle.

The presence of specific antibodies to MAP in serum in apparently healthy as well as diarrheic and/or anemic animals in subclinical stage of infection in the herd was detected by indirect-ELISA. Its sensitivity and rapidity also justify its widespread use as a herd screening test. Screening of 191 cattle by ELISA showed an overall seroprevalence of 37.7%. However, in ­organized farms, seroprevalence varied from 13.3% to 53.1%, whereas in unorganized sectors of three representative districts of West Bengal, it ranged from 5% to 6.7% with one area having an exceptionally high prevalence of 53.3%. As the prevalence rate was quite high, on a retrospective study on the source of animals, it was found that most of the animals in that herd were procured from organized farm(s). The infected animals could have probably spread the infection in a number of animals living nearby. In Uttar Pradesh and Punjab states, 31.9% and 23.3% of seroprevalence of JD in cattle and buffalo were reported, respectively, although lower seroprevalence has been reported in Gujarat (13.39%) [32], Andhra Pradesh (16.26%) [33], and Karnataka (15.14%) [34]. The difference in prevalence pattern of JD could be due to diversity in topography and environment, animal rearing system, and husbandry practices followed in different states of India.

Intradermal skin test evaluates the DTH response 72 h after intradermal injection of PPD and is an indication of the cell-mediated immune response of the animal [35]. In fact, PPDs are undefined crude extracts of Mycobacterium antigens of different origin such as MAP PPDj or Johnin, *M. avium* subsp. *avium* or PPDa or *M. bovis* or PPDb [36]. In this investigation, the overall prevalence of JD by DTH using Johnin was found to be little less compared to ELISA (29.8%), where the prevalence in organized farms varied from 0% to 46.7%, in comparison to the unorganized sector (0-7%). The higher positivity by DTH in organized farms (32.6% vs. 20%) may be due to false positivity as there is possibility of the animals infected with bovine tuberculosis as observed in our earlier study [37]. The results are in corroboration with the findings of ElSayed [38], who also reported less positivity in cattle. The test results through total Johnin test 57/191 (29.8%) are not in agreement with Kalis *et al*. [39], who noted the average percentage of skin test-positive cattle herds ranging from 0% to 17.5%. Considering DTH and ELISA together for interpretation, the positivity of animals to both the tests in organized and unorganized herds was 19.9% and 8%, respectively, with an overall prevalence of 16.8%, whereas 42.5% animals in organized farms were negative to both the tests compared to 70.0% in unorganized farms.

## Conclusions

From this study, it can be concluded that bovine paratuberculosis is prevalent in representative districts of West Bengal with a higher prevalence in organized farms than unorganized sector. The findings also suggest that DTH and ELISA may be used together as herd test to screen cattle for paratuberculosis. However, a detailed study through sampling of a large number of animals is required to find the prevalence pattern of paratuberculosis in unorganized sector of West Bengal.

## Authors’ Contributions

PD and SB conceived and designed the study. JMB and RD carried out sampling and laboratory analysis. AC, PKN, and TKB analyzed and interpreted the data. All authors read and approved the final manuscript.
